# Zirconocene(III) in Organic Synthesis: Does the Ugly Duckling Become a Swan?

**DOI:** 10.3390/ijms27021100

**Published:** 2026-01-22

**Authors:** Jennifer Rosales, Rachid Chahboun, José Justicia

**Affiliations:** Department of Organic Chemistry, Faculty of Sciences, University of Granada, C. U. Fuentenueva s/n, 18071 Granada, Spain; jcrosales@correo.ugr.es

**Keywords:** Zirconocene, titanocene, free radical chemistry, synthesis, methodology

## Abstract

The development of efficient C-C bond-forming reactions remains an important objective in organic chemistry. These reactions are fundamental tools for building complex molecules for diverse applications. Among the various strategies available, radical processes promoted by group IV metals—particularly Ti and its titanocene-type complexes—have shown remarkable versatility and utility in organic synthesis. However, closely related zirconium analogues have historically received less attention and have shown a more limited reactivity profile. Thus, zirconium and its zirconocene-type derivatives have often been regarded as the “ugly duckling” of group IV metal-promoted radical chemistry. Yet recent advances indicate that this “ugly duckling” is beginning to reveal its synthetic potential. In this review, we highlight the main synthetic applications of zirconocene(III) complexes and compare them with those of titanocene(III). Special attention is placed on the generation of reactive zirconocene(III) species and their impact on reactivity. Overall, these developments show how zirconocene(III) chemistry is emerging as a valuable complement to titanocene(III)-based radical transformations, turning our ugly duckling into a beautiful swan.

## 1. Introduction

Organic synthesis—the practice and discipline of constructing intricate organic molecules from simpler precursors—is one of the most vital areas of study in organic chemistry. This field has also profoundly influenced other scientific domains, giving rise to interconnected disciplines such as pharmaceutical chemistry [1,2], life sciences [3], biotechnology, materials science [4], and nanomaterials [5], all of which rely on breakthroughs in synthetic chemistry to obtain the necessary compounds for their research. Many advances in these allied fields would have been unattainable without progress in organic synthesis.

Throughout history, the development of novel synthetic methods and their application in producing diverse molecular structures has been a central responsibility for organic chemists [6]. As a result, a wide array of protocols has been established, ranging from “classic” reactions to those involving organometallics [7], free radicals [8], organocatalysis [9,10], photoredox processes and photocatalytic systems [11,12], thereby broadening the arsenal of synthetic strategies available to chemists worldwide. These approaches have enabled the production of countless compounds in both academic and industrial contexts, including bioactive natural products, agrochemicals, advanced materials, electronic devices, polymers, and other innovations that enhance modern life in academic and industrial settings. Such broad applications underscore the enduring significance and continuous evolution of organic synthesis.

Accordingly, the specific development of selective and efficient C–C bond formation reactions, which enable the synthesis of more complex structures from simpler ones, remains one of the most important objectives in synthetic chemistry [13]. These processes must satisfy several requirements—simplicity, efficiency, and chemo- and/or regioselectivity—to enable short and high-yielding synthetic sequences of target organic molecules. These characteristics, proposed by various authors—such as Hendrickson and his “ideal synthesis” [14]; Trost and Wender, who introduced the concepts of atom and step economy [15,16]; and Baran and Hoffmann with their “redox economy” proposal [17]—are essential for the practical application of such processes in industrial context [18].

**Figure 1:**
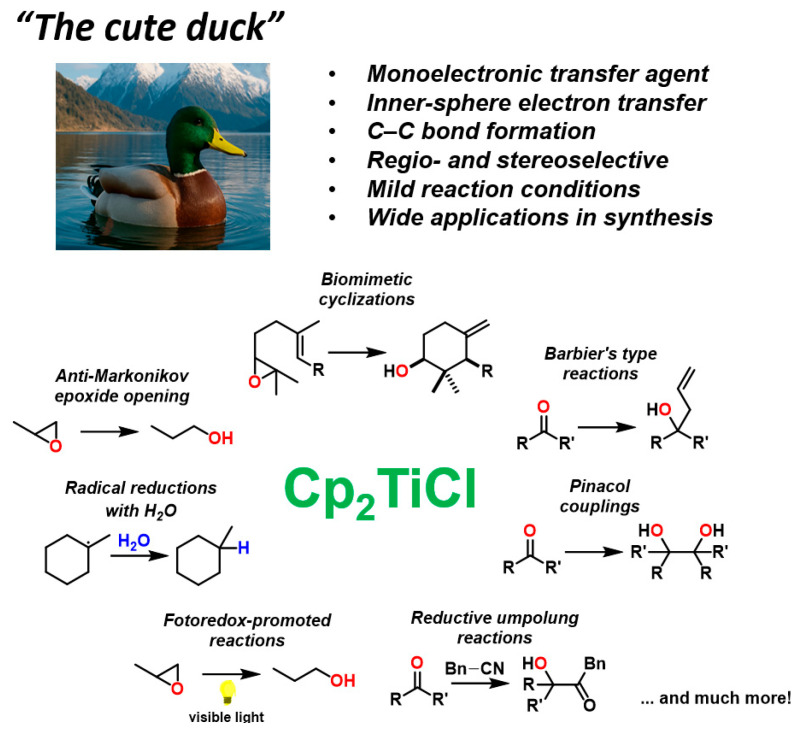
Cp_2_TiCl, the “cute duck” of free-radical chemistry.

In recent decades, transition metal-based methodologies have emerged, exhibiting interesting features that make them compatible with the required specifications for developing efficient synthetic sequences. Among them, Ti(III)-mediated reactions constitute powerful methodologies in organic synthesis, enabling C–C bond formation under mild, room-temperature conditions with broad functional group compatibility (see Figure 1) [19,20,21]. The Cp_2_TiCl (titanocene(III)) complex is generated in situ from commercial Cp_2_TiCl_2_ with powdered Zn or Mn in THF, yielding a mixture of the monomeric Cp_2_TiCl species and the dinuclear species (Cp_2_TiCl)_2_ [22]. Recently, Prof. Gansäuer’s group developed an alternative photoredox procedure [23], opening a new avenue for interesting transformations promoted by Cp_2_TiCl [24,25]. In addition, catalytic methodologies have been reported based on the use of regenerative additives [26,27]. This d^1^ complex, with 15 electrons and a coordination vacancy, facilitates inner-sphere electron transfer through single-electron transfer properties. Titanocene(III) complexes can generate carbon-centered radicals from epoxides [28,29,30], conjugated carbonyls [31,32,33], halides [34,35], allyl carbonates [36], and even ketones and their derivatives [37,38].

The generated radicals have proven useful in various types of reactions, such as different kinds of cyclizations [26,29,39,40,41], employed in the synthesis of several natural products [21,42], including polycyclic compounds of higher complexity with aromatic rings [43], and/or oxygenated functions at different positions of the terpene skeleton [44,45]. Titanocene(III) chemistry has also been applied to the reductive opening of epoxides using a hydrogen atom transfer agent such as 1,4-cyclohexadiene [29], water [46,47], or H_2_ [48]. Moreover, Barbier-type allylation processes promoted by titanocene(III) have also been developed, involving allyl [49], crotyl [35], prenyl [49,50], and propargyl moieties [34], yielding the corresponding products with high regio- and chemoselectivity. Titanocene(III) complexes have further been used in other synthetically valuable reactions, including pinacol couplings of conjugated aldehydes and ketones [33,51], Reformatsky’s type processes [52], coupling reactions of allylic acetates and carbonates with carbonyl compounds using bimetallic systems [53], and the activation of deactivated halocompounds for cyclization and borylation processes [54,55]. Cp_2_TiCl also promotes intra- and intermolecular reductive *umpolung* reactions involving various functional groups, such as ketones, imines, nitriles, Michael acceptors, and others [40,56,57].

In addition to other more specific processes, all the reactions described highlight the remarkable synthetic potential of the titanocene(III) complex. Its utility and versatility have been demonstrated in hundreds of studies over the past 3 decades [19,20,21,24,26,42,57], making it one of the reagents with the greatest capacity to promote diverse stereo-, regio-, and chemoselective transformations under mild conditions. All these features make Cp_2_TiCl an attractive reagent for organic chemists—a beautiful duck in the great lake of organic synthesis, as depicted in Hans Christian Andersen’s famous tale “The Ugly Duckling” [58].

**Figure 2:**
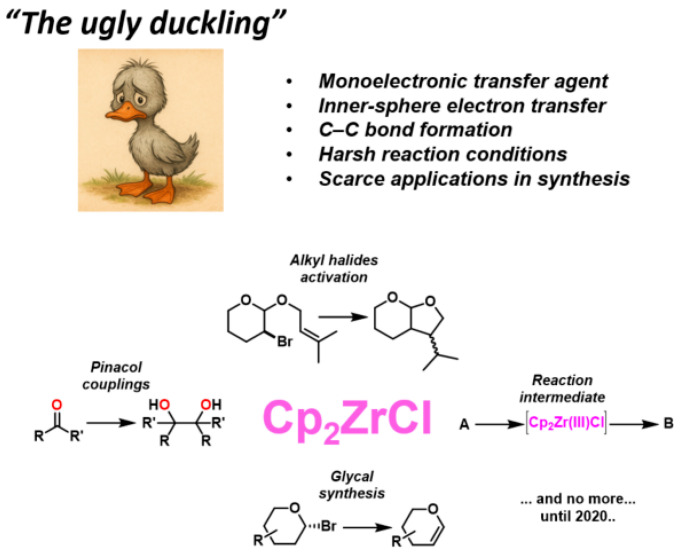
Cp_2_ZrCl, the “ugly duckling” of free-radical chemistry.

In contrast, Cp_2_ZrCl (zirconocene(III)) has received less attention, and its synthetic applications are more limited (see Figure 2). A quick search using the scientific database *SciFinder* indicates that, since 1990, approximately 250 references have been reported for Cp_2_TiCl, and only 38 references for Cp_2_ZrCl. Although this comparison is not entirely rigorous, as many works may fall outside the scope of this search due to their inclusion in the database, it is at least indicative of the significant difference in the use of both complexes in organic synthesis. This observation highlights the complex Cp_2_ZrCl as an “ugly duckling” in the field of group IV metal-promoted free radical chemistry, with a history of limited relevance and use in organic synthesis, despite its promising chemical characteristics.

However, recent findings are changing the perspective on Cp_2_ZrCl, which appears to be evolving from a neglected “ugly duckling” into a relevant “swan” for organic synthesis, as in Andersen’s tale [58]. Based on a logical comparison with the previously discussed reactivity of Cp_2_TiCl, this review aims to provide an overview of its zirconocene analogue, from its first applications at the end of the 20th century to the recently described promising new reactions.

## 2. Cp_2_ZrCl and Its Derivatives in Organic Synthesis

**Figure 3:**
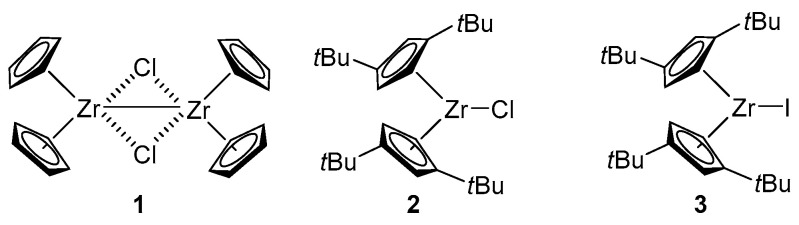
Structures of dimer (Cp_2_ZrCl)_2_ (**1**) and bulky zirconocenes **2** and **3**.

The complex biscyclopentadienyl zirconium(III) chloride (Cp_2_ZrCl) is a deep red solid (although in our hands it consistently appears pink) that is highly sensitive to O_2_ and must be prepared and used under an argon atmosphere. This complex can be generated using diverse reductive methodologies, typically starting from Cp_2_ZrCl_2_ or Cp_2_ZrClH. This reduction requires an important reducing power (E^red^(Cp_2_ZrCl_2_) = –1.70 V vs. SCE) [59], which is more negative than that corresponding to the reduction of Cp_2_TiCl_2_ to Cp_2_TiCl (E^red^(Cp_2_TiCl_2_) = –0.75 V). This represents one of the main disadvantages of this complex, which has limited its use in organic synthesis. Various processes have been described for performing the aforementioned reduction, such as the disproportionation reaction between Cp_2_Zr(CO)_2_ and Cp_2_ZrCl_2_, as described by Floriani et al. for the synthesis and characterization of the complex Cp_2_ZrCl [60]. Other processes and reagents used for the reduction of Zr(IV) to Zr(III) include lithium dialkylphosphides, Na(Hg) or Na-naphthalene [61], Et_3_B [62], and sodium salt of 1,1’-bipyridine [63], etc. This reduction has also been achieved using electrochemical processes [64] and the photolysis of alkylzirconocenes(IV) [65]. Recently, photoredox protocols have also been described [66]. From the original results depicted by Floriani et al. [60], several studies have described Cp_2_ZrCl as the dimer **1** in the solid state and solution (see Figure 3) [61]. However, additional experiments in solution allowed the characterization of closely related derivatives of this complex as monomer species, using phosphine ligands to stabilize the complexes [67,68]. In addition, the introduction of bulky substituents in the Cp rings allowed the synthesis of mononuclear zirconocene(III) complexes, such as compound **2** [69] and the iodide derivative **3** [70], similar to Cp_2_ZrCl. These complexes were characterized by X-ray diffraction, and their structural parameters were established [69,70]. These results indicate that monomeric and dimeric species may coexist in the reaction medium, as with Cp_2_TiCl [22], both participating in the processes described below.

Cp_2_ZrCl also has a d^1^ configuration and a coordination vacancy, enabling its application in inner-sphere electron transfer processes [71]. In addition, it is more oxophilic than its titanocene(III) analog and has shown significant reactivity toward inactivated halides. However, the number of reactions developed with this complex has been very limited over the last 40 years, mainly due to the substantial difficulties involved in generating zirconocene(III) species. As mentioned above, its high reduction potential requires the use of harsh reducing reagents, which are often incompatible with many functional groups. Thus, while related complexes such as Cp_2_ZrCl_2_ and Cp_2_ZrClH have received much attention from synthetic chemists [72], Cp_2_ZrCl has been largely overlooked, becoming the “ugly duckling” in the field of group IV transition-metal-promoted free radical chemistry.

### 2.1. Zirconocene(III) Generated from Strong Reducing Agents

As previously mentioned, the use of the Cp_2_ZrCl complex in organic synthesis has been limited by the difficulty of generating Zr(III) species from various possible sources, especially Cp_2_ZrCl_2_ or Cp_2_ZrClH. Therefore, it is necessary to use reagents with a high reducing capacity, as opposed to the mild and readily available metals—such as Mn or Zn—which are used in the case of Cp_2_TiCl_2_. Alternatives used in studies on the generation and structural determination of zirconocene(III) complexes are usually strong reducing agents and are not always compatible with many functional groups [60,61,62,63,73].

However, there are some examples of successful chemical reactions in which the zirconocene(III) species was generated using one of these strong reducing agents.

**Scheme 1:**
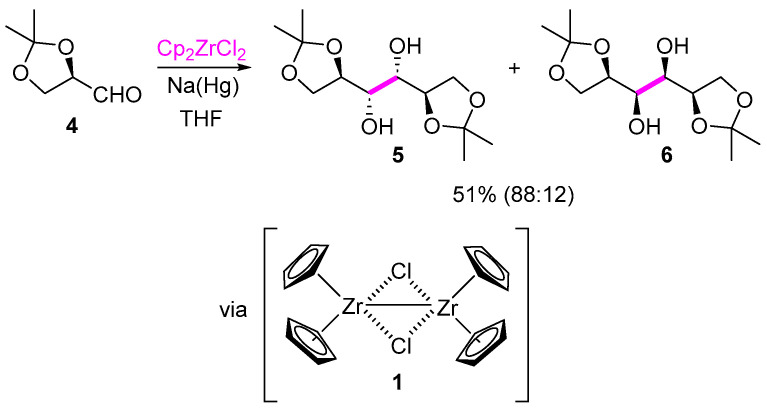
Stereoselective pinacol coupling of aldehyde **4** promoted by Cp_2_ZrCl.

The first example of the application of Cp_2_ZrCl in organic synthesis was described by Barden and Schwartz in 1997 [74]. In this work, the zirconocene(III) species was generated by stirring a solution of Cp_2_ZrCl_2_ in THF with Na(Hg). The generated zirconocene(III) complex was used in the pinacol coupling of aliphatic aldehydes to afford the corresponding vicinal diols with high stereoselectivity and good yields (Scheme 1). This reaction provided an alternative method for synthesizing polyhydroxylated alkyl moieties. In addition, the authors also described the pinacol coupling of acetophenone, yielding a 1:1 mixture of diastereomers in good yield. No examples of aliphatic ketones were reported in this article. This methodology offers advantages over similar reactions promoted by Cp_2_TiCl. As pointed out by Prof. Schwartz, titanocene(III) was unable to perform the pinacolation reaction of aliphatic aldehydes, because it did not have sufficient ability to activate these substrates [75]. This is similar to what occurs in the case of aliphatic halides. However, zirconocene(III) can activate these compounds to generate the corresponding diols, such as the conversion of compound **4** to diols **5** and **6** (see Scheme 1). Another important issue to highlight is the reactive species of zirconocene(III) responsible for the reaction. In this study, Prof. Schwartz proposed that this species is likely (Cp_2_ZrCl)_2_ (**1**), the dimeric form of Cp_2_ZrCl proposed by Floriani et al. [60]. This species reduces aliphatic substrates via one-electron transfer, generating the corresponding radical species. Furthermore, the conversion of dimeric species **1** to monomeric Cp_2_ZrCl, through dissociation or creation of a coordination vacancy, is the rate-limiting step in the process. This suggests the existence of an equilibrium between the two species, analogous to the previously described monomeric and dimeric species of titanocene(III) [22].

**Scheme 2:**
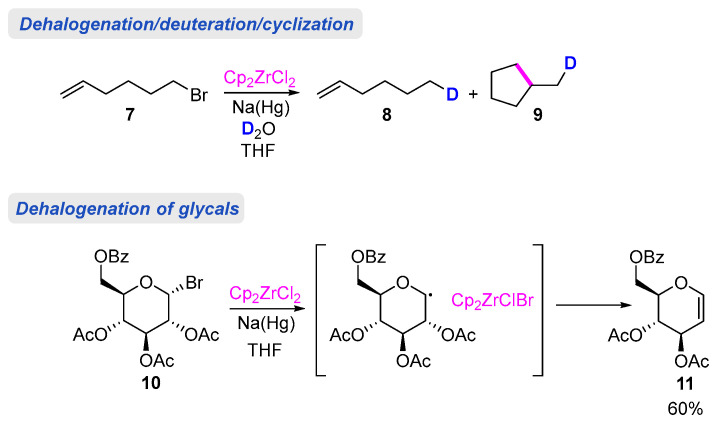
Dehalogenation/deuteration and dehalogenation/cyclization of **7** and dehalogenation in the synthesis of glycal **11** promoted by Cp_2_ZrCl.

Notably, this article not only describes the pinacolation reaction of aliphatic aldehydes but also highlights the ability of Cp_2_ZrCl (either in its monomeric or dimeric form) to abstract halogen atoms from poorly reactive aliphatic halides. This reaction enabled Schwartz et al. to perform dehalogenation/deuteration and dehalogenation/cyclization processes on aliphatic halides, such as **7**, under radical conditions (see Scheme 2). These transformations are based on single-electron transfer from Zr(III) species to alkyl halides, as proposed by Schwartz et al. [76,77].

Another application of zirconocene(III) species generated by reduction with Na(Hg) was the preparation of glycals from the corresponding glucosyl halides. Thus, Prof. Schwartz’s group also described the synthesis of glycal **11** from glucosyl bromide (**10**) in good yield. These results demonstrated the ability of zirconocene(III) species to activate nonreactive alkyl halides and promote the corresponding radical processes [78].

**Scheme 3:**
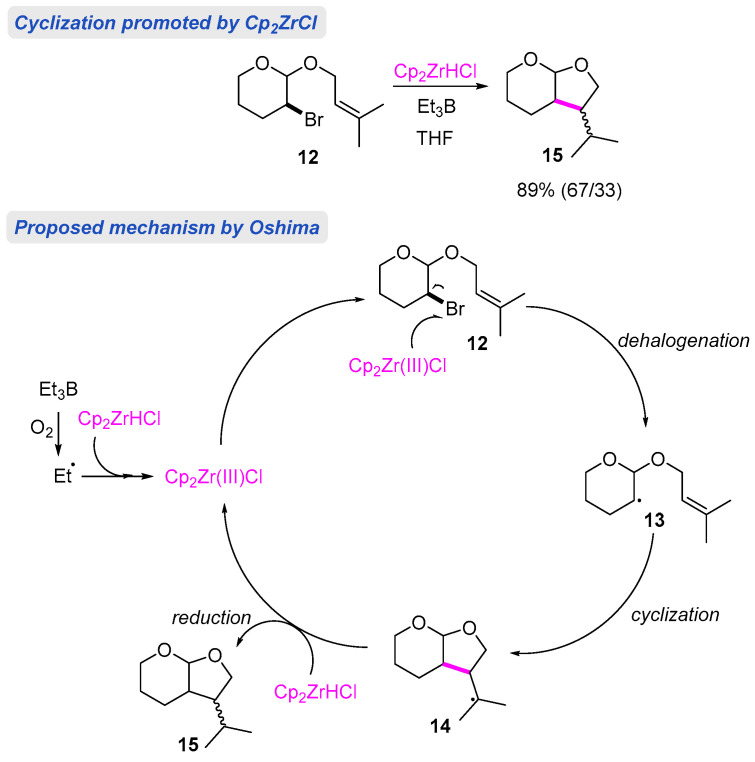
Cp_2_ZrCl-catalyzed cyclization of alkyl halides via dehalogenation.

The interesting ability of the Cp_2_ZrCl complex to activate deactivated alkyl halides, described by Schwartz in the previous examples, was applied by Oshima et al. to develop a series of transformations in which the zirconocene(III) species played a crucial role [79,80]. In these works, Prof. Oshima’s group employed an alternative method for generating Cp_2_ZrCl, avoiding the addition of Na(Hg) amalgam, which has many inconveniences. In this case, they generated the zirconocene(III) species by reducing Cp_2_ZrHCl (Schwartz’s reagent) with Et_3_B (see Scheme 3) [62]. The process started with the generation of an ethyl radical from Et_3_B in the presence of trace amounts of O_2_. Subsequently, the obtained radical homolytically abstracted a hydrogen atom from Cp_2_ZrHCl, yielding the corresponding Cp_2_ZrCl species. In their mechanistic proposal, the authors indicated the formation of a monomeric species of zirconocene(III). However, the existence of an equilibrium with the corresponding dimeric species cannot be ruled out. The generated zirconium(III) species was subsequently applied in radical cyclization reactions of haloacetals, such as **12,** with good yields (Scheme 3). Thus, Cp_2_ZrCl reacted with the corresponding haloacetal **12**, yielding a carbon-centered radical **13** after halogen-atom abstraction by Cp_2_ZrCl. The reaction then proceeded with the addition of the radical formed on the alkene present in **12**, yielding the cyclization product **14**. Finally, the resulting tertiary radical is reduced by HAT from Cp_2_ZrClH to give **15**, regenerating the Cp_2_ZrCl species and closing the catalytic cycle.

**Scheme 4:**
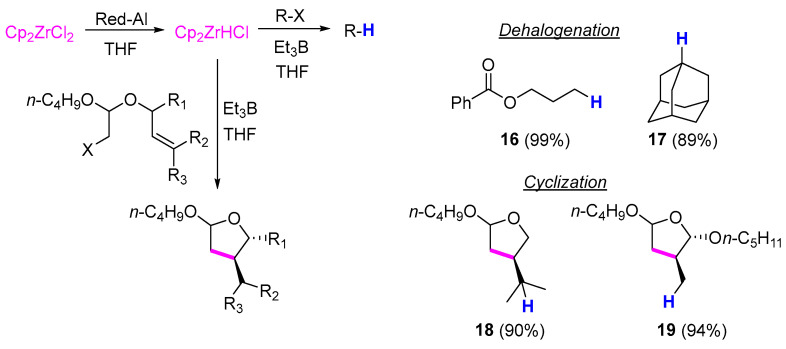
Cp_2_ZrCl-generation from Cp_2_ZrCl_2_ and Red-Al. Application in dehalogenation and cyclization reactions.

Oshima et al. also explored other alternatives for this process, such as the in situ generation of the Schwartz complex (Cp_2_ZrClH) by treating Cp_2_ZrCl_2_ with Red-Al and then applying the aforementioned methodology to generate zirconocene(III) species [81]. In this way, a collection of alkyl halides and haloacetals (preferably with Br or I) were dehalogenated or cyclized, with excellent stereoselectivity and yields (Scheme 4). The presence of Et_3_B prevented the well-known reaction hydrozirconation reaction of the Schwartz’s reagent [72] on the olefin present in the starting compounds.

**Scheme 5:**
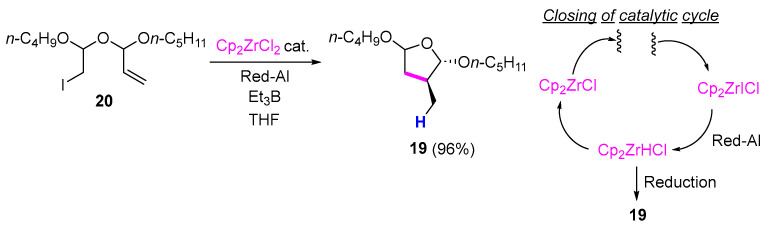
Catalytic proposal for dehalogenation or cyclization of alkyl halides with Cp_2_ZrCl.

Finally, a catalytic variant of this process for dehalogenation and cyclization has been described [81]. For example, acetal iodide **20** (1.0 mmol), Et_3_B (1 mmol), Cp_2_ZrCl_2_ (0.2 mmol), and Red-Al (1.5 mmol) were used to obtain the corresponding cyclization product **19** in 96% yield (Scheme 5). The authors proposed a catalytic mechanism involving two key steps. First, reduction of the intermediate Cp_2_ZrClX (X = Br or I) to Cp_2_ZrClH by Red-Al. Second, the subsequent HAT reduction of the C-centered radical generated during the cyclization by the formed Cp_2_ZrClH, yielding the final product and Cp_2_ZrCl, thus closing the catalytic cycle (see Scheme 5).

**Scheme 6:**
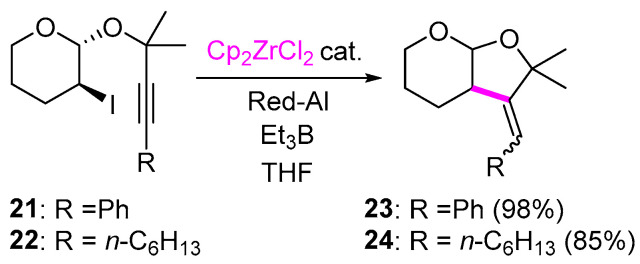
Cyclization of halo-alkynes promoted by Cp_2_ZrCl.

In the same work, Oshima’s group published an extension of this protocol, providing new insights into the cyclization processes promoted by Cp_2_ZrCl [81]. The effect of using alkynes with different degrees of substitution in cyclizations was studied (see Scheme 6). It was observed that the hydrozirconation reaction was significantly faster than radical cyclization with non-terminal alkynes, which is consistent with the high reactivity of Schwartz’s reagent with triple bonds [72]. However, the use of alkynes substituted at the propargylic position, such as **21** and **22**, resulted in the corresponding cyclization products **23** and **24** in excellent yields (see Scheme 6).

Although these studies are interesting examples of the generation and application of Cp_2_ZrCl in organic synthesis, the conditions used remain incompatible with a variety of functional groups, such as epoxides. Thus, developing more similar reactions under the described conditions has not been possible, highlighting the difficulties of zirconocene(III) chemistry.

Other similar studies have been carried out by Oshima’s group, promoting radical processes with Et_3_B and involving zirconocene(III) species. However, these species appear as reaction intermediates, not as the reagents that induce the process. Therefore, they will be discussed in Section 2.2 of this review.

**Scheme 7:**
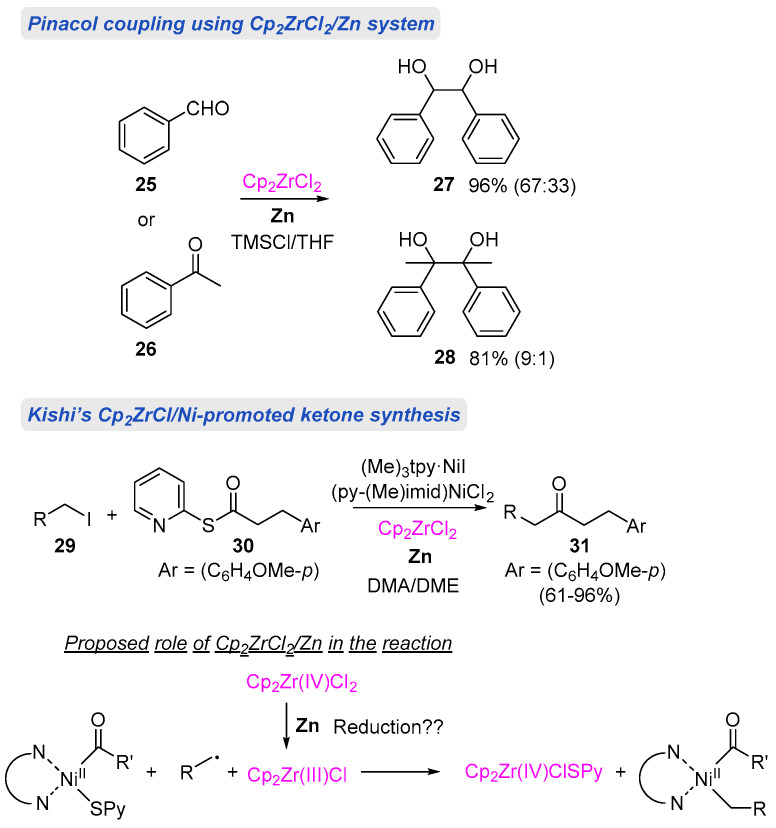
Kamtan’s pinacol coupling and Kishi’s preparation of ketones promoted by Cp_2_ZrCl.

There are no more examples involving the direct generation of Cp_2_ZrCl species using strong reducing agents. However, some proposals have suggested that Cp_2_ZrCl can be obtained using mild reducing agents such as Zn, which is widely used in the context of Cp_2_TiCl but incompatible with Cp_2_ZrCl. It should be remembered that the reduction potential of Cp_2_ZrCl_2_ to Cp_2_ZrCl is −1.7 V [59], while that corresponding to the reduction of Zn(II) to Zn is much lower (−0.75 V) [22]. Thus, Kantam et al. described a pinacol coupling reaction of aldehydes and ketones promoted by Cp_2_ZrCl_2_ in the presence of Mg, which must involve zirconocene(II) species [82]. However, the authors reported that when Zn replaced Mg as the reducing agent, the reaction improved in yield but not in diastereoselectivity, as observed in the examples (see Scheme 7). These results are surprising, since Zn cannot reduce Cp_2_ZrCl_2_ to Cp_2_Zr(II) or to Cp_2_ZrCl.

A similar example is the work of Kishi, Umeara et al., which described a new one-pot process for the preparation of ketones from iodoalkanes and thiopyridine esters catalyzed by a Ni/Cp_2_ZrCl_2_ system, in the presence of Zn as a reductant of Ni and Zr complexes [83]. The authors applied this new process in the context of the synthesis of natural products, such as halichondrins. When describing the proposed mechanism for this reaction, the authors stated that the reaction between Cp_2_ZrCl_2_ and Zn leads to Cp_2_ZrCl, which promotes ligand exchange in the Ni complex (see Scheme 7). This proposal is questionable due to the low reducing capacity of Zn.

**Scheme 8:**
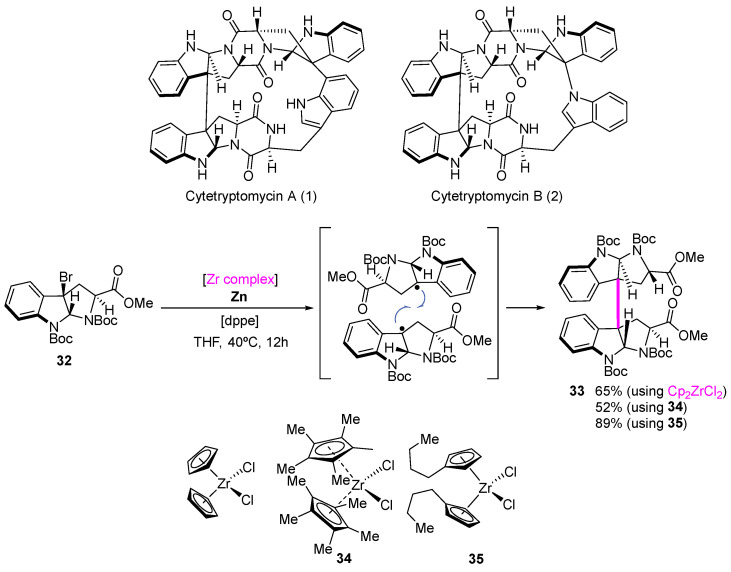
Synthesis of cyctetryptomycin A and B via Cp_2_ZrCl-catalyzed radical dimerization.

Other examples involving the use of Cp_2_ZrCl_2_ and more powerful reducing metals, such as Mn, have not been described as promoted by zirconocene(III) species. Thus, Barrero et al. developed a process for coupling allylic halides mediated by Cp_2_ZrCl_2_/Mn mixtures or Cp_2_TiCl [84]. In both cases, the coupling products, yields, and regioselectivities were quite similar, despite the different mechanisms involved. Thus, in the case of Cp_2_TiCl, it was a radical-based process. However, the authors reported that the Cp_2_ZrCl_2_/Mn mixture is incapable of generating Cp_2_ZrCl, which would promote a similar radical-based process, although via a different mechanism. Therefore, if a reducing agent stronger than Zn, such as Mn, cannot reduce Zr(IV) to Zr(III), it seems unlikely that Zn could do so, as indicated in the previous studies [82,83]. In relation to Kishi’s proposal [83], a subsequent study using the same Ni/Zr system omitted any mention of the reduction of Zr(IV) to Zr(III) by Zn, indicating that its role is unclear [85]. Furthermore, Yamazaki, Qia, and Nakamura et al. proposed a similar process for synthesizing tetrameric tryptophan natural products cyctetryptomycin A and B [86], based on a Zr-catalyzed radical dimerization of alkyl bromides such as **32** (see Scheme 8). This reaction allowed the construction of two consecutive quaternary carbon centers to give **33**. To this end, the authors described the generation of Cp_2_ZrCl and other species from similar zirconocene complexes through Zn reduction. This work provides another intriguing example suggesting the formation of zirconium(III) species by a reducing metal not expected to accomplish this transformation, highlighting the need for further mechanistic and electrochemical studies.

In our opinion, the role of Cp_2_ZrCl_2_ in these processes may be to activate the surface of the metals involved (Zn or Mn), which would be the actual reactants that perform the reaction [87].

### 2.2. Zirconocene(III) Generated as a Reaction Intermediate

As indicated in the previous section, the limitations in the early stages of zirconocene(III) chemistry were due to the difficulty in generating this species, even when using strong reducing agents that were not compatible with some functional groups. Despite its interesting chemical characteristics, this fact significantly limited the possible applications of this complex. However, although not directly, Cp_2_ZrCl can also be obtained in situ as an intermediate in the reaction mechanism. In many cases, this species fulfills an important role in the reactions, owing to its properties as a single-electron reducing agent in radical processes.

**Scheme 9:**
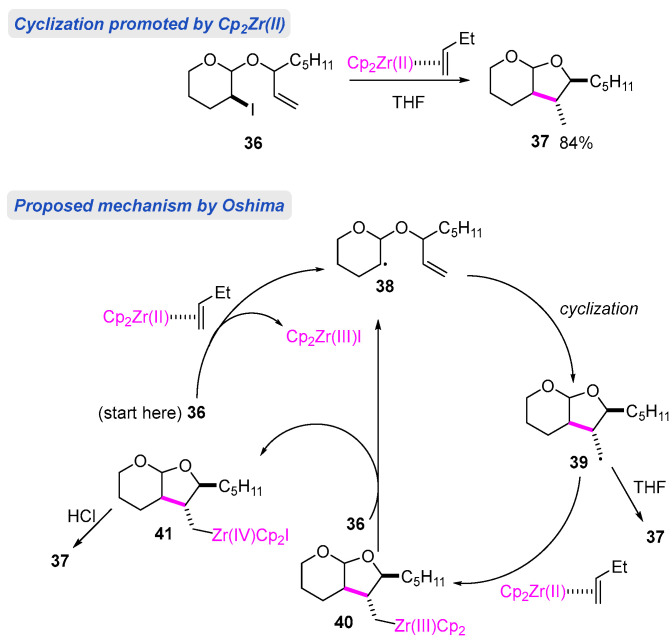
Zirconocene(III)-generated intermediates in the cyclization of β-haloalkyl allyl acetals.

Some examples of this can be found in several papers by Prof. Oshima’s group, which, as we have already indicated, made important contributions to the field of zirconocene(III) chemistry. In 2002, these authors developed a zirconocene–olefin complex-induced reductive radical cyclization of a series of β-haloalkyl allyl acetals [88]. This complex, in which Zr is in the +2-oxidation state, promoted cyclizations through a single-electron transfer reaction (Scheme 9). The mechanism proposed by these authors involves the formation of two species of zirconocene(III) (see Scheme 9). The first species is formed after the reaction of compound **36** with the Cp_2_Zr(II) complex, which promotes the dehalogenation of the alkyl halide, forming Cp_2_ZrX species (X = Cl, Br, I). After the indicated cyclization of radical **38**, the primary radical generated in **39** reacted with the zirconocene(II) complex, yielding an alkylzirconocene(III) intermediate **40**, which reacted again with **36**, generating an alkylzirconocene(IV) (**41**) and **38**, after the abstraction of the corresponding halogen atom. In both cases, the obtained zirconocene(III) species were not reintroduced into the catalytic cycle and did not perform any specific function in the reaction.

**Scheme 10:**
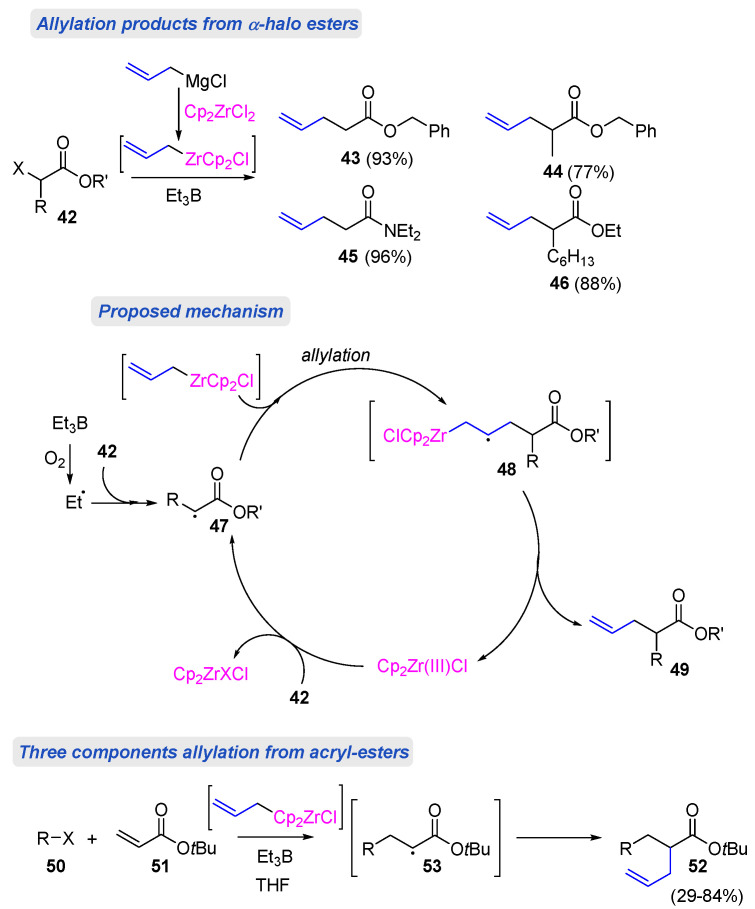
Allylation of α-halocarbonyl compounds promoted by zirconocene species.

In 2004, Oshima et al. described a new and efficient radical allylation process of α-halocarbonyl compounds (mainly esters and amides) (see Scheme 10 for details) [89]. The allylzirconocene(IV) intermediate involved in this reaction was generated by reacting Cp_2_ZrCl_2_ with allylmagnesium chloride. In their mechanistic proposal, the authors reported that the generated carbon-centered radical **47** reacted with the allylzirconocene(IV) after the dehalogenation of the α-halocarbonyl compound **42** by the action of an ethyl radical obtained from Et_3_B, giving the corresponding allyl-addition product **48**. Intermediate **48** involved a radical at the γ position of the carboxyl group and the formation of a new alkylzirconocene(IV). The homolytic fragmentation of **48** generated the final product **49** and Cp_2_ZrCl. The action of the generated zirconocene(III) species closes the catalytic cycle by performing the dehalogenation of the starting compound **42**, again yielding the carbon-centered radical **47** and Cp_2_ZrXCl.

The authors also described a modification of this process, which involves three components in the reaction—allylzirconocene(IV), an alkyl halide (**50**), and an acrylate as **51**—to generate the corresponding α-allylation product **52** (see Scheme 10). In this case, the first step of the proposed mechanism involves the addition of the carbon-centered radical derived from the alkyl halide (again generated from Et_3_B) to the acrylate to yield radical **53**. The subsequent reaction of allylzirconocene(IV) with **53** yielded **52**. The species generated after this second step is Cp_2_ZrCl, which reacts with the starting halide **50**, as in the previous process, thus closing the catalytic cycle after the corresponding dehalogenation.

**Scheme 11:**
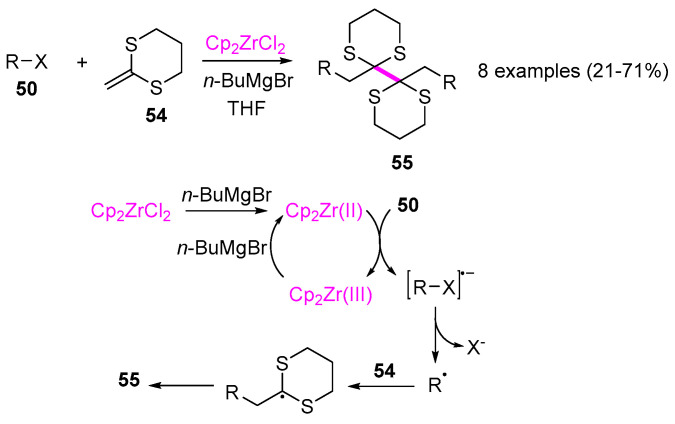
Synthesis of *vic*-bis(dithiane) compounds from tertiary alkyl halides promoted by zirconocene species.

In 2007, Oshima’s group described a new alkylative dimerization reaction of alkyl halides (**50**) and 2-methylene-1,3-dithiane (**54**) mediated by zirconocene(II) to give the corresponding *vic*-bis(dithiane) compounds **55**, which are challenging to access and prepare, with good to moderate yields (see Scheme 11) [90]. Regarding the proposed mechanism, the authors reported that the zirconocene(II) species is generated from Cp_2_ZrCl_2_ and the Grignard reagent *n*-BuMgBr. This species promotes the dehalogenation of halide **50**, generating the corresponding carbon-centered radical, which is subsequently added to 2-methylene-1,3-dithiane (**54**). The formation of this radical drove the process, which is more favorable for tertiary alkyl halides, generating more stable radicals. After the dehalogenation process, the Cp_2_ZrCl species is generated and converted back to Cp_2_Zr(II) by reaction with the Grignard reagent. Therefore, in this case, Cp_2_ZrCl reappears in the process, although its role is relatively minor.

**Scheme 12:**
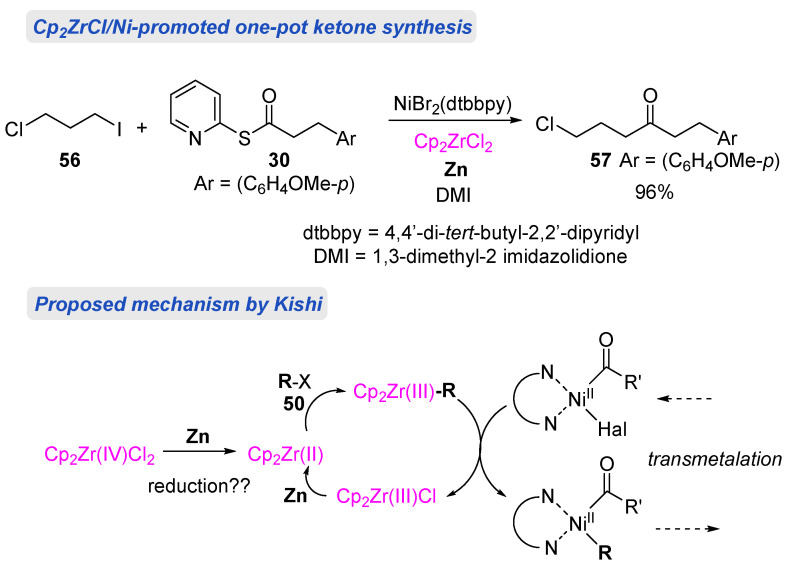
One-pot synthesis of ketones from alkyl iodides and 2-thiopyridine esters.

More recent examples of the generation of zirconium(III) species (mainly Cp_2_ZrCl) as intermediates in several reactions have been described, where these species play a more significant role. One example is the work of Kishi et al., published in 2017. This work described the development of a one-pot preparation of ketones from alkyl iodides and 2-thiopyridine esters using a Zr/Ni system [91], similar to that previously described in this review [83]. The authors efficiently synthesized a variety of ketones, providing relevant synthetic advantages, such as broad functional-group tolerance, good coupling efficiency, high coupling rates, and good stereoselectivity (see Scheme 12). This reaction was used for the efficient and scalable synthesis of natural halichondrin products [92]. The proposed mechanism involves two different catalytic cycles for Ni and Zr, indicating the formation of a Cp_2_ZrCl species in the Zr cycle. Thus, these authors proposed that the reduction of Cp_2_ZrCl_2_ with Zn or Mn leads to the formation of a low-valent Zr(II) species (Cp_2_Zr(II)), which reacts with the alkyl halide **50**, generating the corresponding alkylzirconocene(III). This intermediate participates in a transmetalation process with the Ni intermediate generated in the second catalytic cycle, transferring the alkyl group and generating Cp_2_ZrCl. To close this catalytic cycle, this compound is reduced back to Cp_2_Zr(II) by Zn or Mn reduction (Scheme 12).

**Scheme 13:**
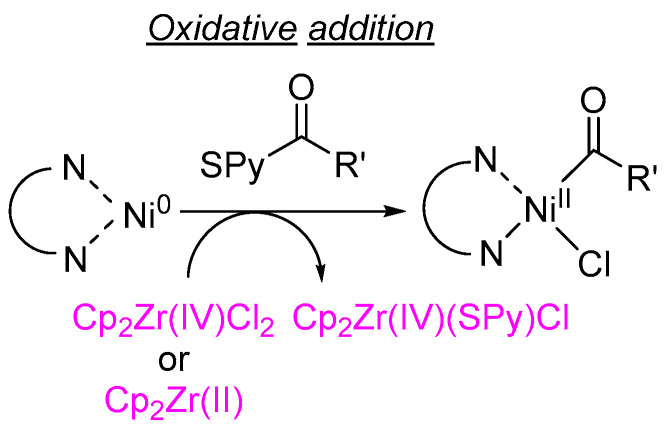
Proposed oxidative addition step by Kishi.

However, this proposal raises some doubts about its validity. The reduction of Zr(IV) to Zr(II) proposed with mild reducing agents, such as Zn or Mn, does not seem possible, as mentioned above [82,83,84,85,86]. Furthermore, the reduction of Cp_2_Zr(III)Cl to Cp_2_Zr(II) with these mild reducing agents is also unlikely. Another question is whether the Cp_2_ZrCl species is formed during the transmetalation process. In the proposed mechanism (see Scheme 13), the oxidative addition of Ni(0) to the 2-thiopyridine ester generates a thiopyridyl group in the new Ni-complex, which is then transferred in the transmetalation step, generating a zirconocene(III) species (Cp_2_ZrSPy), but not Cp_2_ZrCl.The authors did not describe the use of acyl chlorides in the reaction instead of a 2-thiopyridine ester, which would yield the formation of Cp_2_ZrCl. The use of Cp_2_ZrCl_2_ in the oxidant addition step could justify the transfer of a Cl atom to the Ni complex. However, as proposed by the authors, this would not be possible if Cp_2_Zr(II) were involved in this step. Therefore, this mechanistic proposal requires further study.

**Scheme 14:**
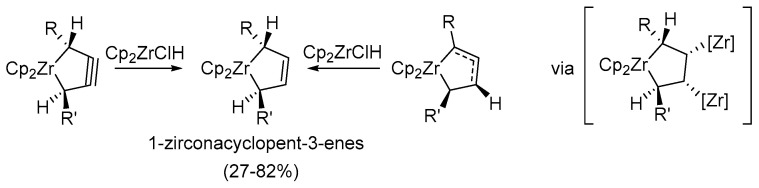
Synthesis of 1-zirconacyclopent-3-enes by (Cp_2_ZrCl)_2_ elimination.

In 2021, Suzuki et al. described a method for synthesizing five-membered zirconacycloalkenes, 1-zirconacyclopent-3-enes, which are formally “hydrogenated” products obtained by the reaction of 1-zirconacyclopent-3-ynes and 1-zirconacyclopenta-2,3-dienes with Schwartz’s reagent (Cp_2_Zr(H)Cl) (see Scheme 14) [93]. Mechanistic studies with deuterated substrates revealed that the reactions proceeded via double hydrozirconation, followed by the elimination of Zr species. In this case, the final step of the reaction involves a Zr–Zr elimination, which generates the dimeric species (Cp_2_ZrCl)_2_, one of the proposed forms of zirconocene(III). This compound is highly labile and readily oxidized to give the μ-oxo-bridged dimer (Cp_2_ZrCl)_2_O [61].

**Scheme 15:**
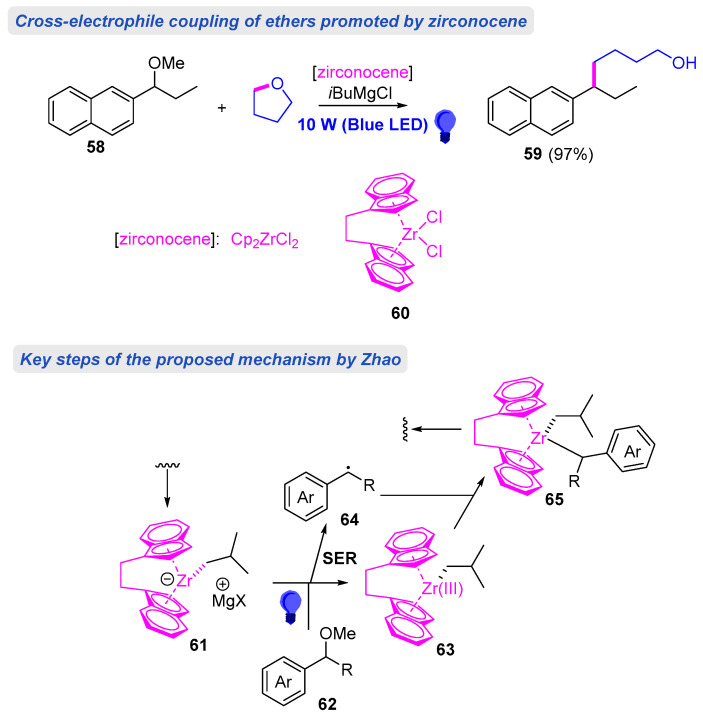
Synthesis of alcohols from photoinduced cross-electrophile coupling of ethers.

In 2024, Zhao et al. reported an efficient method for the reductive coupling of ethers to form C–C bonds with high activity and selectivity [94]. The reaction was catalyzed by photoinduced low-valent zirconocene species. In this way, a diverse range of functionalized alcohols was synthesized through the reaction between benzyl ethers and THF, such as compound **59**, which was obtained from the coupling of methyl ether **58** and THF (Scheme 15). The reaction was studied using Cp_2_ZrCl_2_ as the Zr source. The obtained results were suboptimal because the benzyl ether reduction process competed with the coupling. Therefore, the authors decided to use a bridged *bis*(indenyl)-type zirconocene(IV) complex **60**, which provided excellent results. An extensive mechanistic study of this reaction led the authors to propose a catalytic cycle. The key steps of this mechanism involved the formation of the alkylzirconocene(III) species **63**, after the activation reaction of the C–O bond in the ether **62** by zirconocene(II) complex **61** under light irradiation, generating the corresponding benzylic radical **64** via single electron reduction (SER). This zirconocene(III) intermediate reacts with **64**, yielding the corresponding dialkylzirconocene(IV) **65**, which generates the final product after several subsequent steps.

In our search, we were unable to find other examples of processes in which the species Cp_2_ZrCl or other related species were generated during the reaction and exerted a significant effect on it. The works described in this section, together with those mentioned in the previous one, continue to suggest that Cp_2_ZrCl has a limited presence and relevance in organic synthesis compared to Cp_2_TiCl, giving it the appearance of an “ugly duckling.” However, just as the seemingly irrelevant and unattractive duckling in Andersen’s fairy tale [58] had a happy ending, our protagonist, Cp_2_ZrCl and related species, also seems destined to transform from irrelevance to importance.

### 2.3. Zirconocene(III) Generated by Photochemical Processes: The Ugly Duckling Turns into a Swan

As we indicated in previous sections, the relevance and applications of Cp_2_ZrCl and other similar species have been scarce, despite the interesting chemical characteristics of this type of compound. The main reason for this underuse seems to lie in the difficulty of generating these species efficiently, gently, and compatibly with functional groups from Cp_2_ZrCl_2_ or Cp_2_ZrHCl. Therefore, developing new methodologies for generating zirconocene(III) species could open the possibility of greater application and relevance of these compounds in free radical chemistry and organic synthesis.

In this sense, promising procedures for obtaining zirconium(III) species have also been described based on reactions that do not involve metals. For example, the use of photochemical processes for the generation of Cp_2_ZrCl has been known since the 1980s [64,65], although its application in organic synthesis has been limited until recently. This may be because the original photochemical processes were too aggressive and could affect the functional groups present in the target molecules. However, the introduction of new photochemical processes, which are milder and compatible with a wide variety of functional groups, has given new impetus to zirconocene(III) chemistry [95,96].

**Scheme 16:**
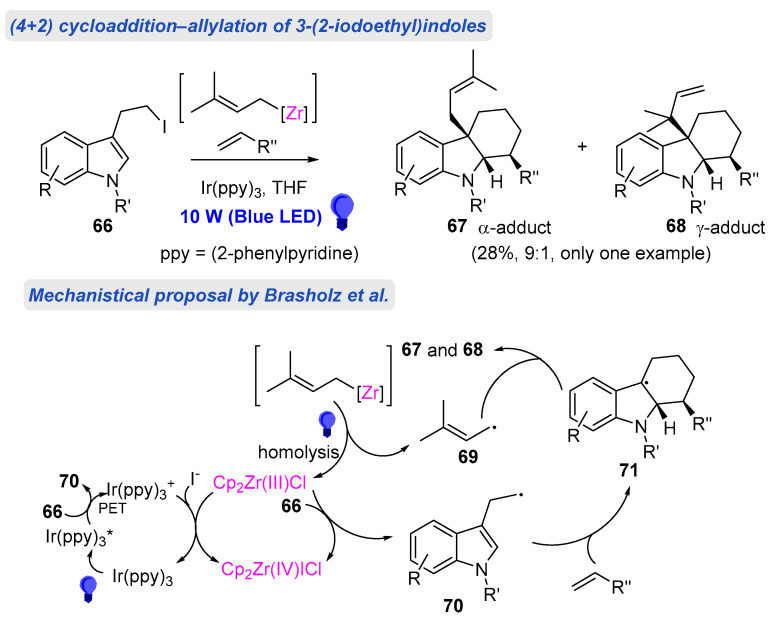
Cycloaddition-allylation of indoles promoted by photolytically generated Cp_2_ZrCl.

In 2017, Brasholz et al. described a light-induced, three-component radical (4 + 2) cycloaddition–allylation process involving 3-(2-iodoethyl)indoles, electron-acceptor-substituted alkenes, and Cp_2_ZrCl(σ-allyl)-type complexes (see Scheme 16) [95,97]. This method provides access to densely functionalized hexahydrocarbazoles such as compounds **67** or **68** in a single transformation, simultaneously forming three carbon–carbon bonds and three adjacent stereocenters. Direct photolysis of allyl zirconocene species triggers the radical pathway, which liberates free allyl radicals and generates Cp_2_ZrCl species.

The proposed mechanism, depicted in Scheme 16, is initiated by the photolysis of the allylzirconocene(IV) complex to generate the carbon π-radical **69** and Cp_2_Zr(III)Cl. Zirconocene(III) intermediates activate the corresponding (iodoethyl)indole **66** by iodine-atom abstraction, yielding Cp_2_ZrICl and the primary carbon radical **70**. This stage, which is key to this mechanistic proposal, can be carried out owing to the ability of zirconocene(III) complexes to promote the homolytic cleavage of inactivated-C(sp^3^)-halogen bonds [74,77]. The generated radical **70** then performs a conjugate addition to the alkene, followed by diastereoselective intramolecular 6-*endo*-trig cyclization to yield benzylic radical **71**. The recombination of radical **71** with the π-radical **69** affords the final products **67** and **68**. Notably, the authors indicate that the role of Ir(ppy)_3_ in the reaction is unclear. They proposed that its triplet-excited form Ir(ppy)_3_* can promote a PET process with the iodoalkane **66**, generating the primary radical **70**, which would be incorporated into the corresponding step of the mechanism. However, the resulting Ir(IV) species would be reduced by Cp_2_ZrCl to regenerate Ir(ppy)_3_, indicating a second function for the zirconocene(III) species. This study is a good example of the application of modern photoinduced methodologies under milder conditions.

**Scheme 17:**
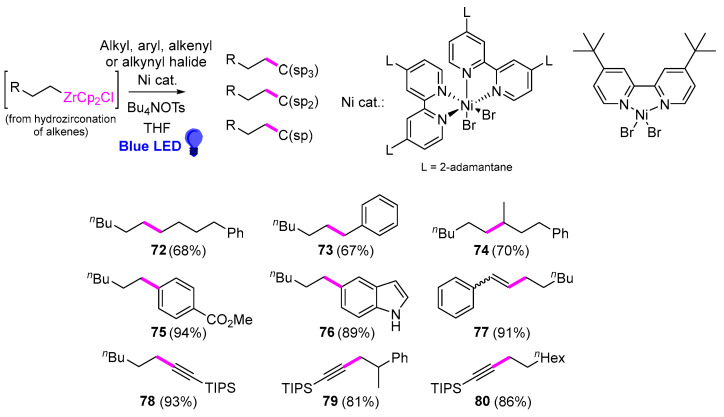
Ni-catalyzed cross-coupling of alkylzirconocenes and diverse halides. Selected examples.

Recently, other similar reactions based on photolytic processes have been described to generate Cp_2_ZrCl from the corresponding alkylzirconocene(IV) complexes. Jiang, Qi et al. described a general nickel-catalyzed cross-coupling protocol mediated by visible light, employing alkylzirconocenes as key intermediates (see Scheme 17) [98].

**Scheme 18:**
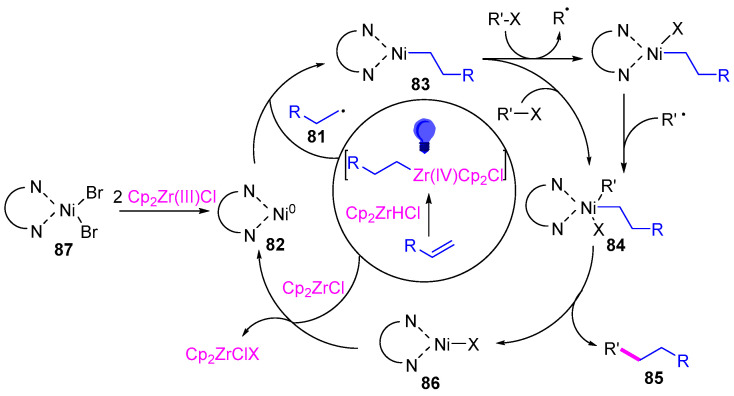
Proposed mechanism for Ni-catalyzed cross-coupling of alkylzirconocenes and halides.

These alkylzirconocenes are generated in situ from terminal or internal alkenes via hydrozirconation and subsequent chain-walking processes. The methodology exhibits broad substrate scope, accommodating a wide variety of organic halides and alkenes while demonstrating excellent functional group tolerance. The reaction performs excellently with different types of alkyl, alkenyl, aryl, and alkynyl iodides and bromides, yielding the corresponding coupling products with good yields under mild reaction conditions and showing scalability, such as examples **72**–**80** depicted in Scheme 17. This strategy provides a valuable platform for the formation of carbon–carbon bonds and constitutes the first example of visible-light-induced cross-coupling using alkylzirconocenes. An extensive theoretical-experimental study was conducted to determine the mechanism of this reaction. Following these studies, a mechanism involving the homolysis of the alkylzirconocene(IV) complex under blue-light excitation was proposed to generate the corresponding alkyl radical 81 and Cp_2_ZrCl (Scheme 18). The obtained alkyl radical **81** reacts with the Ni(0) complex **82** to produce an alkyl–Ni(I) intermediate **83**, which evolves to give the final product **85**. The new Ni(I) complex **86** generated after the reductive elimination of **84** is reintegrated into the catalytic cycle by reduction to Ni(0) complex **82** by Cp_2_ZrCl, which is transformed into Cp_2_ZrClX. Previously, the authors also proposed that Cp_2_ZrCl reduces the original Ni(II) complex **87** to Ni(0). Therefore, these would be the functions of the zirconocene(III) species in the proposed mechanism.

**Scheme 19:**
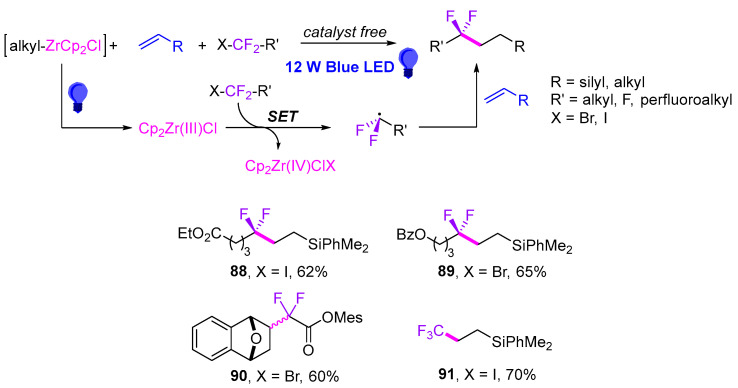
Fluoroalkylation of alkenes promoted by photolysis of alkylzirconocenes.

Another interesting example of the generation of Cp_2_ZrCl through a photolytic process is the work published by Zhang et al. in 2022 [99]. In this article, the authors describe the development of a difluoroalkylation reaction of alkyl- and silyl-alkenes with inactivated difluoroalkyl iodides and bromides (see Scheme 19). The process was promoted by Cp_2_ZrCl, formed after visible light irradiation of an alkylzirconocene. In this way, the authors were able to obtain a variety of difluoroalkylated compounds useful in organic synthesis, such as examples **88**–**91** (Scheme 19). The reaction was also applicable to activated difluoroalkyl, trifluoromethyl, perfluoroalkyl, monofluoroalkyl, and nonfluorinated alkyl halides, thereby establishing a broadly applicable approach for the controlled synthesis of fluorinated compounds.

The proposed mechanism indicates that the key step is the generation of Cp_2_ZrCl by photolysis of an alkylzirconocene (IV) under blue LED irradiation (12 W). This species is formed in situ by hydrozirconation of the starting alkyl- or silyl-alkene with Schwartz’s reagent. Cp_2_ZrCl reacts with the difluoroalkyl halide, causing homolytic cleavage of the C-X bond, which generates the corresponding difluoroalkyl radical and Cp_2_ZrClX. The generated radical then adds to the starting olefin to form the corresponding alkylation product. Therefore, the process proceeds under mild conditions, is compatible with various functional groups, and provides good yields. Furthermore, the previous generation of the corresponding alkylzirconocene(IV) avoids possible incompatibility between the alkenes used and Cp_2_ZrHCl.

The examples described are approaches that allow the use of Cp_2_ZrCl, which is mildly generated and compatible with many functional groups, enabling its application in various reactions. However, they have a significant drawback: the need to prepare the corresponding alkyl or alkenyl-zirconocenes, usually by adding Schwartz’s reagent to alkenes or alkynes. This introduces additional steps into the sequence and does not meet the requirements of atom and steps economy in modern organic synthesis [14,15,16,17]. Therefore, developing methodologies that allow the direct and simple generation of Cp_2_ZrCl or similar compounds from accessible reagents remains essential for efficient free radical chemistry reactions.

**Scheme 20:**
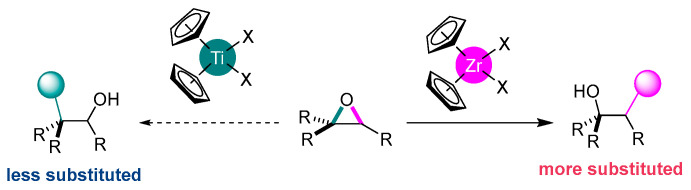
Reverse regioselectivity compared to titanocene(III) catalysis.

**Scheme 21:**
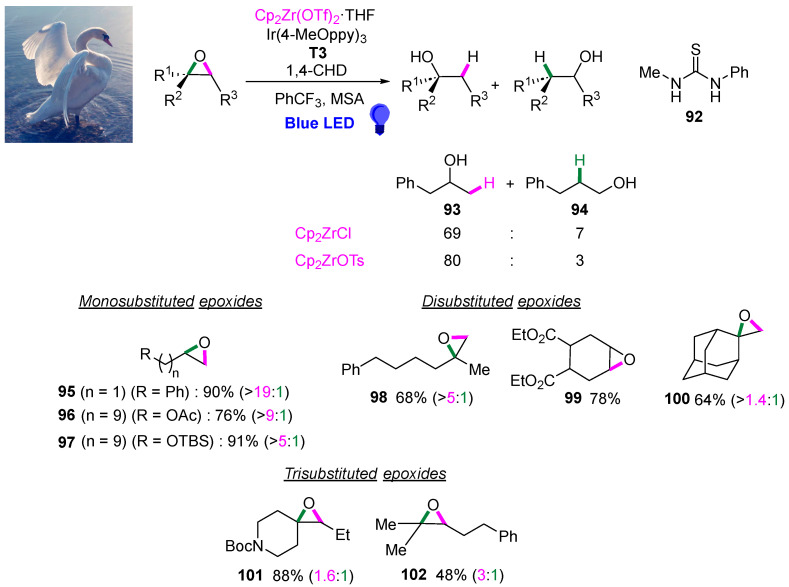
Zirconocene(III)-catalyzed epoxide opening. General reaction and selected examples.

Fortunately, this important goal has recently been achieved, enabling the development of a series of interesting reactions promoted by Cp_2_ZrCl and its derivatives, thanks to the excellent work of Ota and Yamaguchi’s group [96]. Thus, in 2022, these authors published an unprecedented zirconocene(III)-catalyzed epoxide ring-opening activated by photoredox catalysis using blue LEDs [66]. This reaction stands out due to its inverse regioselectivity (see Scheme 20), arising from the higher oxophilicity of zirconocene compared to titanocene, which makes the ring-opening more exothermic. Unlike titanocene-based methods, which usually generate anti-Markonikov alcohols, it favors the formation of more substituted alcohols (Markonikov regioselectivity) via less stable radicals [19,20,21,22]. Although the reaction occurs when Cp_2_ZrCl is used, the regioselectivity is not entirely satisfactory (69:7 for compounds 93 and 94, see Scheme 21). Therefore, the authors studied other similar zirconocene complexes, such as Cp_2_ZrOTf, which yielded improved regioselectivity (80:3 for the same compounds, Scheme 20).

This reaction operates under mild conditions and is compatible with a wide variety of functional groups, even in complex natural products. The protocol has demonstrated remarkable versatility (Scheme 21), proving effective with mono-, di-, and tri-substituted epoxides 95–102, as well as natural product derivatives, such as estrone, cholesterol, allose, and capsaicin. Its effectiveness has been confirmed with terminal epoxides 95–97 bearing diverse functional groups and with 1,2-disubstituted epoxides of various ring sizes (e.g., 98 and 100). However, trisubstituted epoxides such as 102 yielded modest results. Other relevant applications of this reaction include intramolecular cyclizations triggered by the addition of the generated radicals to alkenes (as previously described for Cp_2_TiCl-promoted processes) and a remarkable synthesis of benzylidene acetals from the ring opening and cyclization of epoxy-benzyl ethers after a 1,5-HAT.

An extensive mechanistic study was conducted to establish the various aspects of this reaction. The authors confirmed that the process follows a radical pathway through experiments with a “radical clock” or the addition of TEMPO, a well-known radical scavenger. The regioselectivity of the process was further supported by computational calculations, which showed that ring opening—endergonic with titanocene—is extremely exergonic with zirconocene. This thermodynamic difference correlates directly with the significantly lower activation free energies for zirconocene, validating the design hypothesis based on the higher oxophilicity of zirconium. The characteristics of the transition state in zirconocene reactions—with shorter C–O scissile bonds and lower spin density on the generated radical—suggest an “early” or “reactant-like” transition state, explaining the pronounced influence on regioselectivity. Another relevant aspect of this protocol is the crucial role of thiourea-type reagents, particularly thiourea **92**. This compound plays an essential role in enhancing regioselectivity, complementing the effect of changing the metal center. It is postulated that a zirconocene(III)-thiourea complex is fundamental to the irreversible opening step and that a possible interaction between thiourea, the starting zirconocene(IV), and the epoxide may occur. This interaction suggests that thiourea and the epoxide might bind to zirconocene(IV) before its reduction to the active zirconocene(III) species, potentially influencing regioselectivity.

**Scheme 22:**
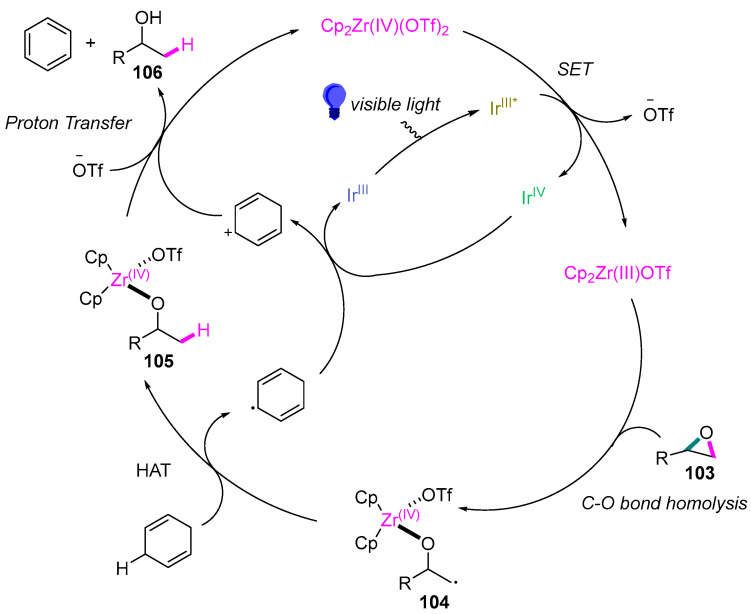
Proposed mechanism for zirconocene(III)-catalyzed epoxide opening.

Scheme 22 illustrates the proposed mechanistic approach.

The zirconocene(III) complex was obtained by reducing Cp_2_Zr(OTf)_2_ with the corresponding excited Ir(III) photocatalyst, generating Cp_2_ZrOTf. This compound interacts with epoxide **103**, promoting its homolytic opening and generating the less substituted radical via intermediate **104**. Subsequent reduction of this radical by 1,4-CHD [26] affords intermediate **105**, and cleavage of the O-Zr bond by addition of TsO- gives the corresponding Markovnikov alcohol **106** and regenerates Cp_2_Zr(OTf)_2_, closing the catalytic cycle. A clear disadvantage of this process compared to those described for Cp_2_TiCl is that the product corresponding to the reduction of the intermediate radical is always obtained, without the appearance of compounds derived from a mixed disproportionation step, as proposed by Justicia et al. [100]. Therefore, alternative conditions that prevent early radical reduction are necessary to broaden the scope of this reaction.

**Scheme 23:**
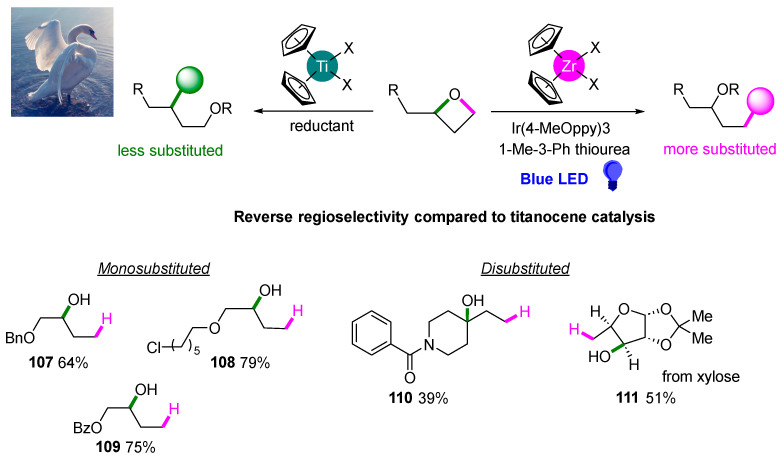
Zirconocene(III)-catalyzed oxetane opening. General reaction and selected examples.

Applying the same reaction conditions, these authors also described a highly regioselective radical opening of oxetanes catalyzed by Cp_2_ZrOTf (see Scheme 23) [101]. In this case, the Markovnikov opening product is again obtained via the less stable radical, providing a process that is complementary to that described by Gansäuer et al. [102]. Again, the reaction proceeds under mild conditions, is compatible with a variety of functional groups, as shown in examples **107**–**111** (Scheme 23). This reaction was also used to synthesize six-membered benzylic acetals from the corresponding benzylic ethers. The mechanistic issues discussed for the epoxide opening process [66] also apply to this work. Furthermore, this process also yields intermediate radical reduction compounds, without mixed disproportionation processes that generate double bonds in the final step of the reaction, as described by Gansäuer’s group [102].

**Scheme 24:**
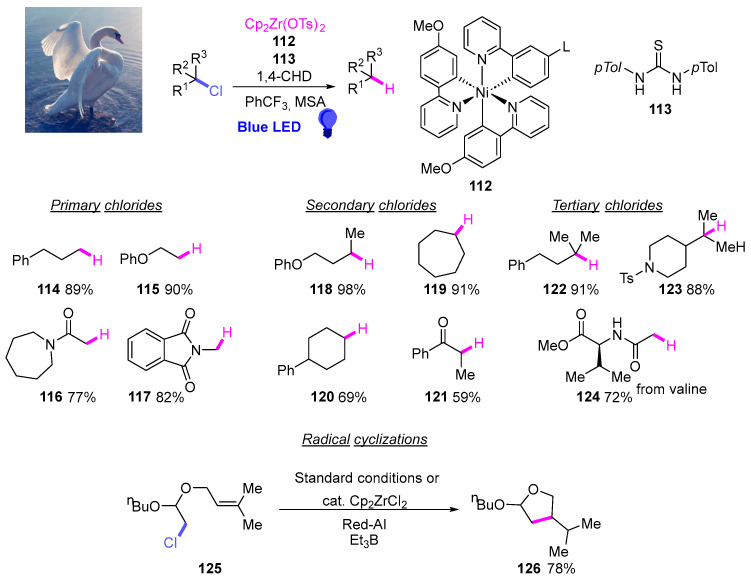
Zirconocene(III)-catalyzed dehalogenation of inactivated halides. General reaction and selected examples.

As indicated in the previous sections, one of the effective reactions that Cp_2_ZrCl can perform is the dehalogenation of unactivated C-X bonds. Therefore, Ota and Yamaguchi’s group applied the photoredox generation methodology of Cp_2_ZrCl to promote reactions involving this type of substrate. Alkyl chlorides, for example, present significantly higher bond dissociation energies (BDEs) (84 kcal/mol for C-Cl) than alkyl bromides or iodides, but these compounds are attractive due to their abundance, easy preparation, and ubiquitous presence in natural products. However, the existing SET approaches for the dehalogenation of these substrates often require strongly reducing conditions, or their application is restricted to sterically accessible primary and secondary chlorides. To overcome these drawbacks, in 2023, Ota, Yamaguchi et al. studied the generation of carbon-centered radicals from unactivated alkyl chlorides catalyzed by Cp_2_ZrCl, including sterically hindered tertiary alkyl derivatives (Scheme 24) [103]. The authors hypothesized that zirconium, by forming a robust Zr–Cl bond (127 kcal/mol), thermodynamically facilitates halogen-atom transfer (XAT) from inactivated alkyl chlorides, making the process more exergonic and viable. Thus, the application of the reaction conditions previously described [66] allowed the development of a novel zirconocene photoredox catalytic protocol, promoted by the reduction of Cp_2_ZrX_2_ to zirconocene(III) by an excited iridium photocatalyst, and subsequently by XAT.

This reaction was applied with excellent results to the dechlorination of primary, secondary, and tertiary alkyl chlorides, yielding the corresponding reduced products **114**–**124** (Scheme 24), even in complex compounds such as cholesterol, glucofuranoside, or diosgenin. Remarkably, radical cyclizations examples, such as the cyclization of alkyl chloride **125** to furan derivative **126**, were also described (see Scheme 24). The protocol was also extended to the photochemical borylation of inactivated alkyl chlorides—a challenging but synthetically valuable transformation. As a result, valuable alkyl boronic esters were efficiently obtained. The proposed mechanism for this process is analogous to that of epoxide opening. In this case, the reaction can be efficiently performed using either Cp_2_ZrOTs or Cp_2_ZrCl. The alkyl radical generated is also reduced by 1,4-CHD addition. In the case of borylation, the addition of the boron group appears to be faster than the HAT from 1,4-CHD. Notably, this new reaction provides an operational advantage for Cp_2_ZrCl over Cp_2_TiCl, which has been rarely used to promote XAT processes of nonactivated halides.

The generation of a carbon-centered radical during the dehalogenation process described in the previous study could also lead to alternative transformations. One example is the dimerization of the radical species obtained. Several precedents exist in titanocene(III) chemistry [104], such as the reductive homocoupling of allyl halides. Various radical-based and visible-light methods for benzyl halides homocoupling have also been reported [105]. However, benzyl chlorides remain challenging substrates for visible-light-mediated homocoupling. In this context, Ota, Yamaguchi et al. developed an innovative catalytic protocol that generates carbon radicals from benzyl chlorides using zirconocene and photoredox catalysis [105]. This method operates through an XAT mechanism facilitated by the formation of a robust Zr–Cl bond (127 kcal mol^−1^), whose reactivity does not depend on strongly reducing conditions or redox potential.

**Scheme 25:**
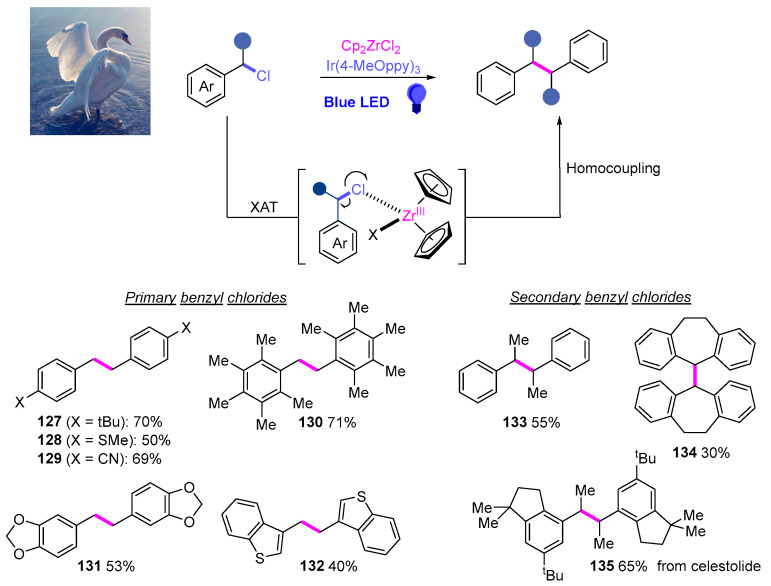
Zirconocene(III)-catalyzed homocoupling of benzyl chlorides. General reaction and selected examples.

**Scheme 26:**
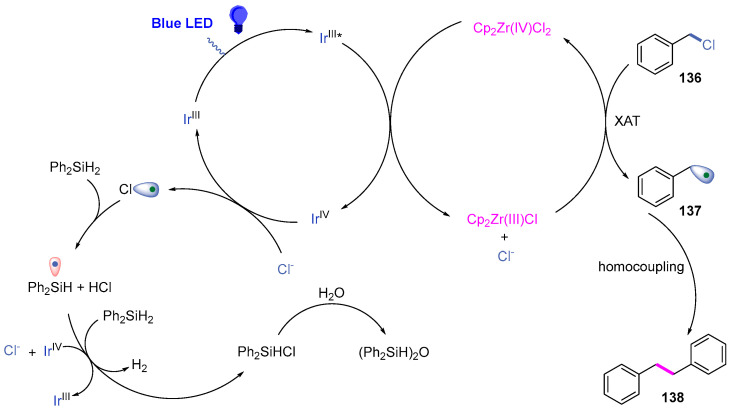
Proposed mechanism for zirconocene(III)-catalyzed homocoupling of benzyl chlorides.

The authors used previously described protocols and obtained better results when the Cp_2_ZrCl species was generated. In addition, the HAT agent 1,4-CHD was replaced by Ph_2_SiH_2_ to avoid the formation of undesired reduction byproducts. This reaction was applied to the reductive homocoupling of a wide range of benzyl chlorides, yielding the corresponding dimers **127**–**135** (Scheme 25), under mild conditions and showing compatibility with a variety of functional groups. The practicality of the catalytic protocol was demonstrated by the successful synthesis of pharmaceutical agent derivatives in a single step. The reaction was also tested on other substrates, showing inconsistent outcomes with allyl chlorides and low or no yields with benzyl bromide and benzyl fluoride, highlighting the specificity of the method for benzyl chloride activation. Scheme 26 illustrates the proposed mechanism, which closely resembles that described in previous studies. The only new feature is the inclusion of Ph_2_SiH_2_, used to reduce the Cl radical generated after the reduction of Ir(IV) to Ir(III) by the chloride anion formed upon conversion of Cp_2_ZrCl_2_ to Cp_2_ZrCl. The use of 1,4-CHD, a more efficient HAT agent, may reduce the benzyl radical intermediate.

The group of Ota and Yamaguchi also reported a reaction similar to the previous one, involving an unprecedented α-defluorination of fluorinated carbonyl compounds catalyzed by Cp_2_ZrCl [106]. The reaction is highly selective and does not affect other functional groups, such as bromo-, chloro-, or trifluoromethyl-substituted arenes. In the presence of γ-terpinene as the HAT agent and (*n*BuCp)_2_ZrCl, the authors obtained the corresponding α-defluorinated products of ketones, esters, and amides. This protocol was also applied to the defluorinative alkylation of various esters and amide alkene derivatives.

**Scheme 27:**
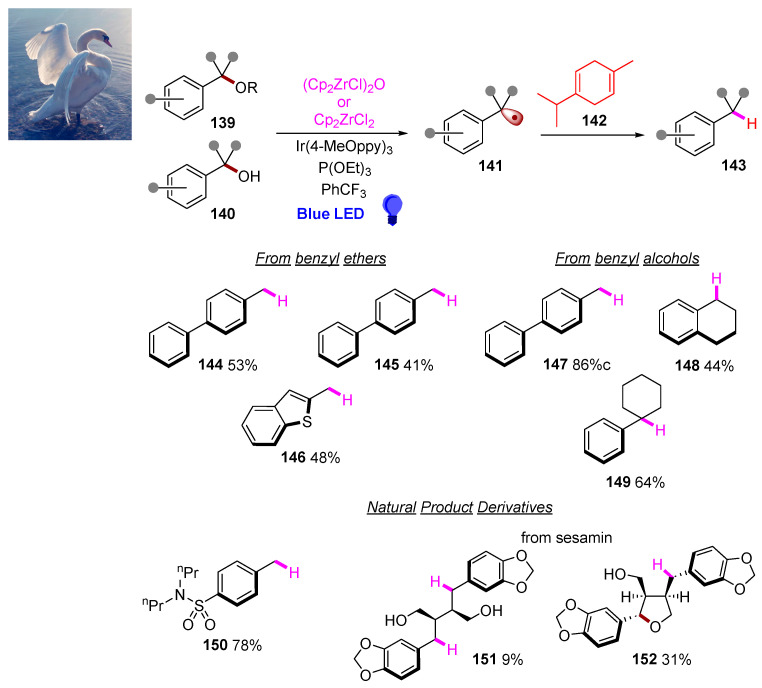
C-O bond cleavage in benzylic alcohols and ethers catalyzed by Zr(III).

The homolytic cleavage of the C–O bond in alcohols and ethers generates carbon radicals, enabling access to products not attainable through polar methods. However, this transformation is challenging due to the high bond dissociation energies involved. Advances in photoredox catalysis have overcome this limitation. Moreover, C–O bond cleavage in alcohols has been achieved through titanium catalysis without pre-functionalization. Several researchers (Sato, Barrero, Zheng, Shu, Wu, and Fleischer) have reported catalytic protocols for the deoxygenation of alcohols [107]. In contrast, the functionalization of ethers remains less explored due to their more demanding pre-activation. Recently, Ota and Yamaguchi’s group described an efficient zirconocene–photoredox system for the deoxygenation of alcohols and ethers, using γ-terpinene (**142**) as the HAT agent (Scheme 27) [107].

This reaction was studied using various zirconocene complexes to generate reactive Zr(III) species. Thus, Cp_2_ZrCl afforded the corresponding deoxygenation products in moderate yield. However, the dimeric species (Cp_2_ZrCl)_2_O gave improved results and was therefore selected as the precursor for generating the active Zr(III) species. The reaction was tested on a range of benzylic ethers and alcohols, including complex structures such as benzofurans and tetrahydrofurans derivatives, yielding the corresponding deoxygenated products, such as **144**–**149** (see Scheme 27). The protocol was also effective for the deoxygenation of acetophenones. Furthermore, the methodology was successfully applied to bioactive molecules, affording the corresponding deoxygenated derivatives **150**–**152**. The results showed that the catalyst exhibits high selectivity toward benzylic bonds. Mechanistic studies suggest that Cp_2_ZrCl is the active species responsible for the transformation, which proceeds via a radical pathway.

## 3. Conclusions and Perspectives

This review has summarized the main synthetic applications of zirconocene(III) complexes from their first applications in the 1990s to the present, emphasizing their comparison with the more established titanocene(III) systems. Despite their potential, the limited methods available for generating zirconocene(III) species have restricted their use in synthesis.

Recent advances, particularly photoredox methods under visible light, now offer milder and more efficient alternatives to the classical approach based on strong reducing agents. The emergence of Cp_2_ZrCl as a reactive intermediate further broadens the synthetic scope of zirconocene chemistry. From the pioneering studies of Schwartz to the modern work of Ota and Yamaguchi, zirconocene(III) chemistry has evolved from an overlooked “ugly duckling” into a promising “swan”, an efficient tool for radical transformations.

Future research should focus on extending these protocols to new catalytic systems, including oxidative conditions and natural product synthesis. Exploring transformations analogous to those developed for titanocene(III) will allow zirconocene chemistry to reach its full synthetic potential. The swan is flying.

## Data Availability

No new data were created or analyzed in this study. Data sharing is not applicable to this article.
